# Optimal Time Frequency Fusion Symmetric Dot Pattern Bearing Fault Feature Enhancement and Diagnosis

**DOI:** 10.3390/s24134186

**Published:** 2024-06-27

**Authors:** Guanlong Liang, Xuewei Song, Zhiqiang Liao, Baozhu Jia

**Affiliations:** 1 Naval Architecture and Shipping College, Guangdong Ocean University, Zhanjiang 524088, China; 15014603924@stu.gdou.edu.cn (G.L.); tianya0218s@126.com (X.S.); 2 Technical Research Center for Ship Intelligence and Safety Engineering of Guangdong Province, Zhanjiang 524088, China; 3 Guangdong Provincial Key Laboratory of Intelligent Equipment for South China Sea Marine Ranching, Zhanjiang 524088, China

**Keywords:** bearing fault diagnosis, signal feature enhancement, optimal time frequency fusion SDP, deep convolutional neural network (DCNN)

## Abstract

Regarding the difficulty of extracting the acquired fault signal features of bearings from a strong background noise vibration signal, coupled with the fact that one-dimensional (1D) signals provide limited fault information, an optimal time frequency fusion symmetric dot pattern (SDP) bearing fault feature enhancement and diagnosis method is proposed. Firstly, the vibration signals are transformed into two-dimensional (2D) features by the time frequency fusion algorithm SDP, which can multi-scale analyze the fluctuations of signals at minor scales, as well as enhance bearing fault features. Secondly, the bat algorithm is employed to optimize the SDP parameters adaptively. It can effectively improve the distinctions between various types of faults. Finally, the fault diagnosis model can be constructed by a deep convolutional neural network (DCNN). To validate the effectiveness of the proposed method, Case Western Reserve University’s (CWRU) bearing fault dataset and bearing fault dataset laboratory experimental platform were used. The experimental results illustrate that the fault diagnosis accuracy of the proposed method is 100%, which proves the feasibility and effectiveness of the proposed method. By comparing with other 2D transformer methods, the experimental results illustrate that the proposed method achieves the highest accuracy in bearing fault diagnosis. It validated the superiority of the proposed methodology.

## 1. Introduction

Bearings are widely used in industrial production. The primary function of bearings is to support and reduce friction in rotating parts to ensure the regular operation of mechanical systems. In complex severe working environments, bearings often suffer damage that can eventually result in bearing faults [1]. If faults are not detected in a timely manner, it may lead to equipment downtime, production stoppages, or even human casualties and significant economic losses [2]. Timely detection and diagnosis of bearing faults are critical to ensuring equipment safety [3]. Due to the harsh working environment, background noise often submerges bearing fault features, making bearing fault features difficult to extract and reducing the accuracy of bearing fault diagnosis. Therefore, enhancing the fault features of the acquired signals is critical in order to improve the accuracy of bearing fault diagnosis.

Now, numerous studies have been researched by scholars to enhance signal fault features. These studies mainly enhance fault features in the signal or suppress background noise through signal processing [4], machine learning [5,6], deep learning [7], and other methods. Pang et al. [8] proposed an improved empirical Fourier decomposition (IEFD) method. This method can effectively suppress the over-decomposition of noise and faulty pulses; Zhu et al. [9] proposed an enhanced empirical Fourier decomposition method. Lei et al. [10] proposed a rolling bearing fault diagnosis method based on variational modal decomposition and weighted multidimensional feature entropy fusion. The method exhibits good convergence and noise resistance. Mao et al. [11] proposed a method that combines variational mode decomposition (VMD) and K-singular value decomposition (K-SVD) to enhance bearing fault features. Li et al. [12] proposed a method for early weak fault diagnosis in rolling bearings based on a multilayer reconstruction filter. The method has the advantage of reducing the influence of noise and decomposing modal adaptation. Although the above method successfully enhances bearing fault features, 1D signals still have limitations in terms of highlighting critical feature information of complex signals. Transforming 1D signals into 2D images is a beneficial processing method that effectively emphasizes weak fault features [13]. This transformation allows the 1D signal to be intuitively mapped onto a 2D image, making it more conducive to highlighting potential fault features, and providing richer feature information [14]. Youcef et al. [15] proposed a new method that combines convolutional neural network (CNN) and vibration spectral imaging (VSI) to classify bearing faults. This method offers strong generalization and noise resistance. However, a multi-scale signal analysis is needed, which may result in poor performance when identifying complex signals. Wang et al. [16] proposed a rolling bearing fault diagnosis method based on the whale optimization algorithm, variable modal decomposition (VMD), and graph attention network (GAT). The method optimizes the parameters of the variational mode decomposition (VMD) by adaptively selecting the best parameters and choosing signal components with high correlation for reconstruction. Finally, the graph structure data are constructed using the K nearest neighbors (KNN) method and input into the GAT fault diagnosis model for fault diagnosis. The method exhibits strong noise reduction capability, and the fault diagnosis model demonstrates robust stability across various bearing fault states. However, the method has the disadvantage of high computational complexity. Tang et al. [17] proposed a feature ranking method for enhancing bearing fault features based on feature ranking using an optimal class distance ratio (FROCDR). The technique first utilizes multi-scale analysis and variational modal decomposition to process the vibration signals at various scales and frequency bands. Subsequently, it transforms the processed signals into images using the symmetric dot pattern (SDP) transformation method. Finally, the FROCDR method obtains the best feature subset as input for the random forest classifier in bearing fault diagnosis. The method helps extract multi-scale features of the signal, enhances the expression of fault features, and exhibits high stability. However, the method suffers from low computational efficiency in selecting SDP parameters. Li et al. [18] proposed a rolling bearing fault feature enhancement method that combines the adaptive symmetric dot pattern and density-based spatial clustering of noise applications (ASDP-DBSCAN). The method first transforms the vibration signal into a symmetric dot pattern (SDP) and then optimizes the SDP image parameters using the Hill function with a genetic algorithm. Finally, a modified DBSCAN algorithm generates clustering templates to reduce noise interference and enhance fault features. The method offers high noise immunity and adaptive parameter selection. However, it also presents drawbacks, such as high computational complexity and a need for multi-scale analysis of complex signals.

From the above research projects, the 1D to 2D method by SDP is an effective method for enhancing fault features. SDP can directly transform 1D signals into 2D images to display signal features. The computational efficiency is higher compared to many other image transformation methods. However, the traditional SDP method has the disadvantage of difficulty in selecting parameters and fault information is limited. Therefore, this paper proposes an optimal time frequency fusion SDP method for enhancing bearing fault features. Considering that the absence of multi-scale analysis in the SDP image method results in buried signal fault features and decreased diagnostic accuracy, the method employs a time frequency fusion algorithm to analyze signal fluctuations at minor scales across multiple scales. It maps the signal analysis results at various scales onto a 2D image. At the same time, by considering the influence of multiple parameters of SDP on the effectiveness of feature extraction, the optimal SDP parameters are adaptively determined using the bat algorithm, which can amplify the distinctions in the images among various fault states of the bearings, enhance the fault features, and improve diagnostic accuracy. The extracted SDP features are input to the DCNN network to achieve fault diagnosis of bearings. This method resolves difficulties in parameter selection, the lack of multi-scale analysis for complex signals, high computational complexity, and achieves enhanced bearing fault features. The main contributions of this paper are as follows:

- An optimal time frequency fusion SDP-based signal processing method is designed. The method can effectively display the signal analysis results on various scales, enhance the representation of the signal fault features, and amplify the distinctions between different bearing faults.
- A new fault feature enhancement technique is proposed. The technique can analyze signal time domain and frequency domain feature information at various scales, achieving the fusion of time-domain and frequency-domain features. which enhances the fault signal features and improves the diagnostic accuracy of the model.
- A novel framework for diagnosing rolling bearing faults has been established. The bearing fault diagnosis model is established by extracting optimal time frequency fusion-based SDP image feature information using a DCNN.

This study is structured as follows: Section 2 presents the optimal time frequency fusion SDP method. Section 3 details the rolling bearing fault diagnosis framework in this paper. Section 4 demonstrates the feasibility of the method presented in this paper through experiments. Section 5 verifies the effectiveness and superiority of the proposed method through comparison experiments.

## 2. Optimal Time Frequency Fusion SDP

### 2.1. SDP Transform Method of Vibration Signals

**Figure 1:**
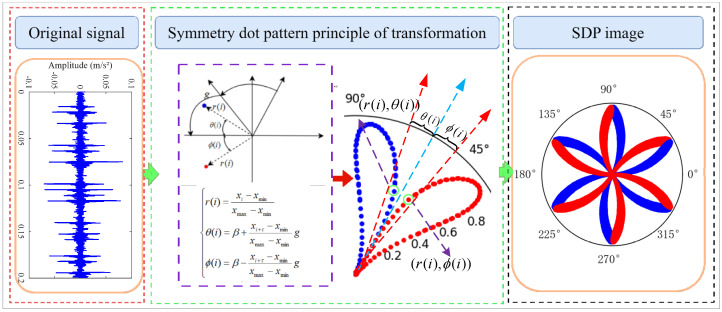
Principle of vibration signal to SDP transformation.

The symmetric dot pattern (SDP) method is a signal representation method that transforms 1D time-domain signals into 2D polar coordinate images using a formula [19]. Formula (1) represents the acquired vibration signals, which are transformed into points (r(i),θ(i)) and (r(i),∅(i)) in polar coordinates using Formula (2). Figure 1 illustrates the transformation principle.

(1) $$X=\{x_{1},x_{2},\ldots ,x_{i},x_{N}\}$$

where X is the 1D vibration signal acquired, $x_{1}$ is the first signal data point, and N is the length of the signal.

(2) $$\left\{ \begin{array}{l} r(i)=\frac{x_{i}-x_{\min}}{x_{\max}-x_{\min}}\\ \theta (i)=\beta +\frac{x_{i+\tau }-x_{\min}}{x_{\max}-x_{\min}}g\\ \varphi (i)=\beta -\frac{x_{i+\tau }-x_{\min}}{x_{\max}-x_{\min}}g \end{array}\right.$$

where r(i) is the polar coordinate radius of the *i*th signal, $x_{i}$ is the amplitude of the *i*th data point, $x_{min}$ is the smallest amplitude of the 1D signal, $x_{max}$ is the largest amplitude of the 1D signal, τ is the time-delay parameter, *g* is the angular amplification factor, β is the angle of rotation of the mirror symmetry plane, β=360n∕m, n=(1,2,⋯,*m*), m is the number of mirror planes, θ(i) is the angle of rotation along the β-clockwise, and ∅(i) is the angle of rotation along the β-counterclockwise clockwise.

### 2.2. Time Frequency Fusion SDP Transformation Method of Vibration Signals

**Figure 2:**
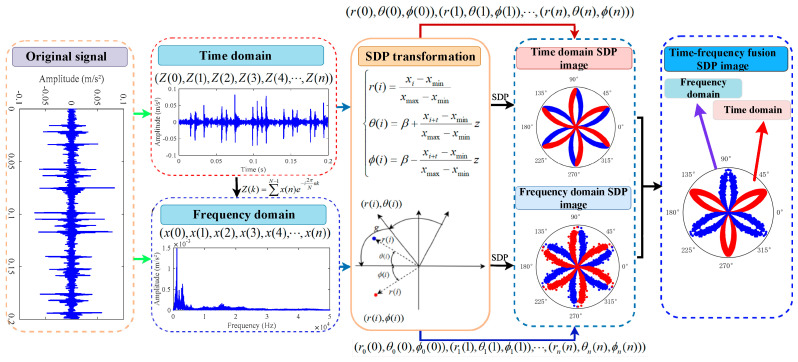
Principle of vibration signal to time frequency fusion SDP transformation.

The time frequency fusion SDP analysis method is a fusion transformation technique that combines the time domains and frequency domains of a 1D signal using SDP analysis. (See Figure 2). Firstly, the signal in the time domain is transformed into a signal in the frequency domain using Formula (3) [20].

(3) $$X(k)=\sum_{n=0}^{N-1}x(n)e^{-i\frac{2\pi }{N}kn}$$

where X is the Fourier transformed coefficient, X(*k*) is the *k*th Fourier transformed coefficient, x is the input signal, $e^{-i\frac{2\pi }{N}kn}$ is the complex term, and x(n) is the nth input signal point.

### 2.3. Optimal Time Frequency Fusion SDP Transformation Method of Vibration Signals

**Figure 3:**
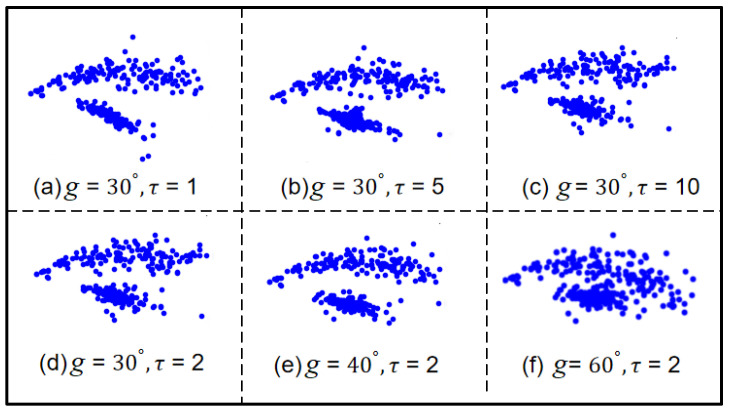
SDP image petals for different values of τ and *g*.

The selection of τ and *g* values in SDP image transformation significantly impacts the fault features. This paper proposes an optimization method for SDP image parameter settings based on the adaptive bat algorithm to find the optimal SDP parameters. In SDP image transformation, τ controls the sparseness of the petals, while *g* controls the degree of opening and closing of the petals. τ takes values in the range 1~10, and *g* takes values in the range $20^{{}^{\circ}}$~$60^{{}^{\circ}}$. If the values of τ and *g* are too large or too small, it may cause the fault features to be buried and reduce the model identification accuracy. As illustrated in Figure 3, for different τ and *g* values of SDP image petals, with different parameters, the images are very different. Since the bat algorithm has global and local search capabilities, the τ and *g* parameters are, therefore, optimized by using the bat optimization algorithm.

#### 2.3.1. Bat Optimization Algorithm

**Figure 4:**
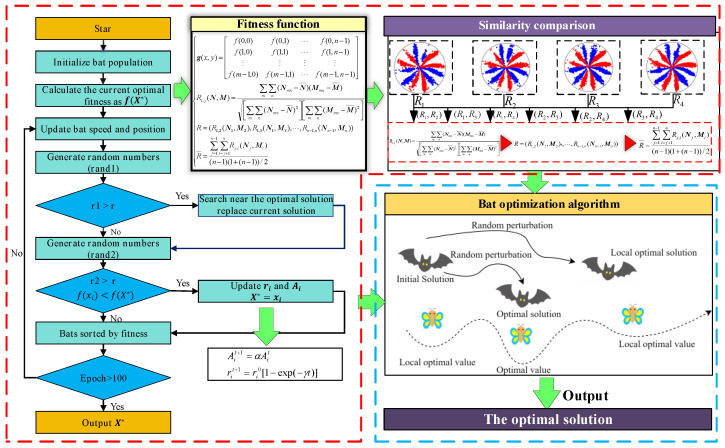
Schematic diagram of the bat algorithm.

The bat algorithm is an optimization algorithm that simulates the process of an individual bat searching for prey [21]. The algorithm is used first of all to initialize the population of bats, determining individual optimal solutions from each bat’s current position, and then to update the position of the bats. Updating the position and speed can be represented by the mathematical model in Formulas (4)–(6). Subsequently, a random number r1 in the range [0, 1] is randomly generated, r1∈[0,1], if $r1>r_{i}$, and a new solution is generated near the optimal solution by Formula (7). If $r1<r_{i}$, generating a random number r2, r2∈[0, 1], if $r2<A_{i}$, the fitness function value is calculated and the fitness function value is less than the fitness function value of the optimal solution, the optimal solution is replaced, and, at the same time, the acoustic loudness $A_{i}$ and frequency $r_{i}^{t+1}$ are updated according to Formulas (8) and (9). Eventually, the above steps are repeated to find the optimal solution individual to the space. The specific steps of the bat algorithm are illustrated in Figure 4.

(4) $$F_{i}=F_{\min}+(F_{\max}-F_{\min})*\beta$$

(5) $$v_{i}^{t}=v_{i}^{t-1}+(x_{i}^{t}-X_{*})F_{i}$$

(6) $$x_{i}^{t}=x_{i}^{t-1}+v_{i}^{t}$$

(7) $$x_{new}=X_{*}+\varepsilon A^{t}$$

(8) $$A_{i}^{t+1}=\alpha A_{i}^{t}$$

(9) $$r_{i}^{t+1}=r_{i}^{0}[1-\exp(-\gamma t)]$$

where $F_{i}$ is the frequency of the sound wave emitted by the *i*th bat in a population, β is a random number and β∈ [0, 1], $X_{*}$ is the current optimum solution, $x_{new}$ is the new solution, ε is a random number and ε∈ [0, 1], $A^{t}$ is the average loudness of the bats in the population as a whole, α is the attenuation coefficient and α∈ [0, 1], γ is the impulse enhancement coefficient, and $r_{i}^{0}$ is the frequency at the initial time of the *i*th bat.

#### 2.3.2. Fitness Function

This paper calculates the average similarity between the optimal time frequency fusion SDP images generated under different bearing fault states. The optimal time frequency fusion SDP method is selected based on the image parameter with the lowest average similarity. The specific steps are as follows:

The initial step is to transform the 1D signals of the various states of the bearing into 2D images using the optimal time frequency fusion SDP method. Next, two SDP images (N and M) in different states are selected sequentially and digitized. The digital matrix can be represented by Formula (10), and its similarity R(N,M) can be determined using Formula (11) [22]. Finally, the similarity between all compared images is counted using Formula (12), and the average similarity value is calculated using Formula (13).

(10) $$g(x,y)=\left[\begin{array}{cccc} f(0,0) & f(0,1) & \cdots & f(0,n-1)\\ f(1,0) & f(1,1) & \cdots & f(1,n-1)\\ \vdots & \vdots & \ddots & \vdots \\ f(m-1,0) & f(m-1,1) & \cdots & f(m-1,n-1) \end{array}\right]$$

where (0,0) is the pixel coordinate origin, (1,0) is the coordinate of the first row of pixels in column zero, and so on for other pixel point coordinates. f(x,y) is the pixel value of the pixel point with coordinates (x,y). n×m is the total number of pixel points.

(11) $$R_{i,j}(N,M)=\frac{\sum_{m}\sum_{n}\left(N_{mn}-\bar{N})(M_{mn}-\bar{M}\right)}{\sqrt{\left[\sum_{m}\sum_{n}{\left(N_{mn}-\bar{N}\right)}^{2}\right]\left[\sum_{m}\sum_{n}{\left(M_{mn}-\bar{M}\right)}^{2}\right]}}$$

where N is the digital matrix of image N, $\bar{N}$ is the average value of pixel points of image N, M is the digital matrix of image M, $\bar{M}$ is the average value of pixel points of image M, i is the bearing state i image, and j is the bearing state j image.

(12) $$R=(R_{1,2}(N_{1},M_{2}),R_{1,3}(N_{1},M_{3}),R_{1,4}(N_{1},M_{4})\cdots ,R_{z-1,z}(N_{z-1},M_{z}))$$

where $R_{1,2}(N_{1},M_{2})$ is the similarity between the bearing state 1 image and the bearing state 2 image, $N_{1},{\;M}_{2}$ are the digitized matrices for bearing state 1 and bearing state 2, respectively, and z is the bearing state z.

(13) $$\bar{R}=\frac{\sum_{e=1}^{z-1}\sum_{f=e+1}^{z}R_{e,f}(N_{e},M_{f})}{(z-1)\times z/2}$$

where z is the number of bearing states, $R_{e,f}(N,M)$ is the similarity between the bearing state e image and the bearing state f image, and $\bar{R}$ is the average similarity between all states.

## 3. The Proposed Method

### 3.1. Feature Extraction Method

**Figure 5:**
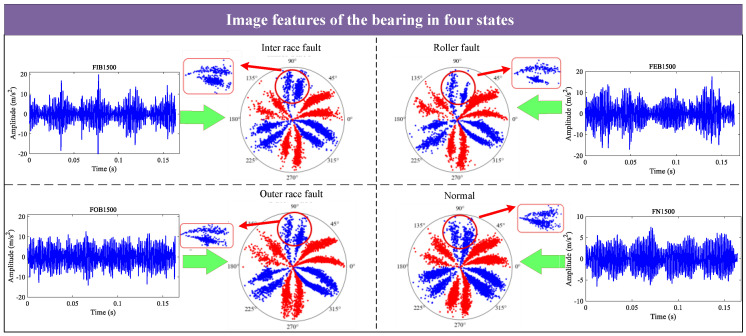
SDP features of the bearing in four states.

The optimal time frequency fusion SDP method can combine multi-scale signals within 1D signals on the SDP image, enabling the image to capture signal features fully. For example, Figure 5 illustrates the image features of the bearing in four states (inner race fault, roller fault, outer race fault, and normal).

Figure 5 illustrates that the SDP image expresses the signal features of the bearing in different states, such as the sparseness of the petals and the degree of opening and closing. Therefore, the bearing state can be determined from the sparseness of the petals and the degree of opening and closing in the SDP image.

### 3.2. Deep Convolutional Neural Network Diagnosis Model

**Figure 6:**
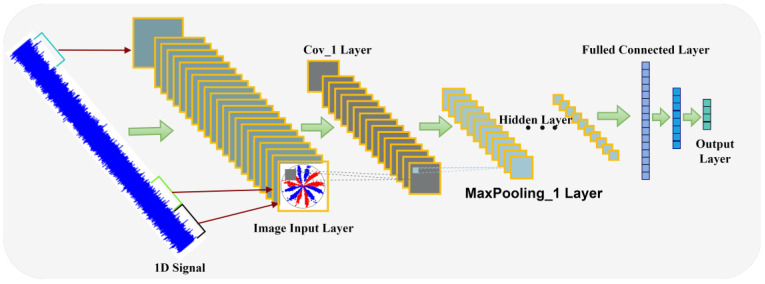
Diagnostic model.

A convolutional neural network (CNN) usually consists of a convolution layer, a pooling layer, a fully connected layer, an activation function, etc. [23]. A CNN has powerful feature extraction and learning capabilities, while a deep convolutional neural network (DCNN) learns more complex feature representations and improves the model’s generalization compared to a simple convolutional neural network. Therefore, this paper adopts a DCNN as the diagnostic model. The model is illustrated in Figure 6. The DCNN uses a convolution layer for feature extraction and nonlinear variation through the ReLU activation function [24], as described by Formulas (14) and (15).

(14) $$X_{i,j}^{l+1}=\sum_{j=1}^{L}\sum_{i=1}^{m}\left(X_{i,j}^{l}\times w_{i,j}^{l}\right)+b$$

(15) f(x)=max(0,x)

where $X_{i,j}^{l+1}$ is the jth eigenvalue of the ith eigenmap in the lth layer of the network, L is the convolution kernel, $w_{i,j}^{l}$ is the weight parameter, and b is the bias value.

The pooling layer reduces image dimensions, preserves image feature information, and enhances neural network training speed [25]. Since average pooling averages the features within the window, it may lead to the loss of some important information. The maximum pooling layer is able to preserve the most significant image feature information and discard the unimportant details, thus, improving the model’s training effect. Therefore, the maximum pooling layer is chosen to pool the image. The maximum pooling formula is illustrated in Formula (16).

(16) $$MaxPooling(I)=\max_{i,j}\;I(i,j)$$

where i and j are denoted as positions within the pooling window and ${max}_{i,j}(I(i,j))$ is denoted as the maximum operation on the value of input data I at all positions $I\left(i,j\right)$.

The role of the fully connected layer in a neural network is to integrate the abstract features output from the convolution and pooling layers and splice them into a 1D vector as input to the network [26]. In addition, by introducing the Softmax function of Formula (17), the fully connected layer can normalize these integrated features so that the network can output a confidence level for each category, enabling effective classification [27].

(17) $$soft\max{\left(Z\right)}_{i}=\frac{e^{Z_{i}}}{\sum_{j=1}^{K}e^{Z_{j}}}$$

where $Z_{i}$ is the *i*th element of the input vector, *e* is the base of the natural logarithm, and $\sum_{j=1}^{K}e^{Z_{j}}$ refers to the exponential sum of all elements of the input vector species.

### 3.3. The Proposed Method Framework

**Figure 7:**
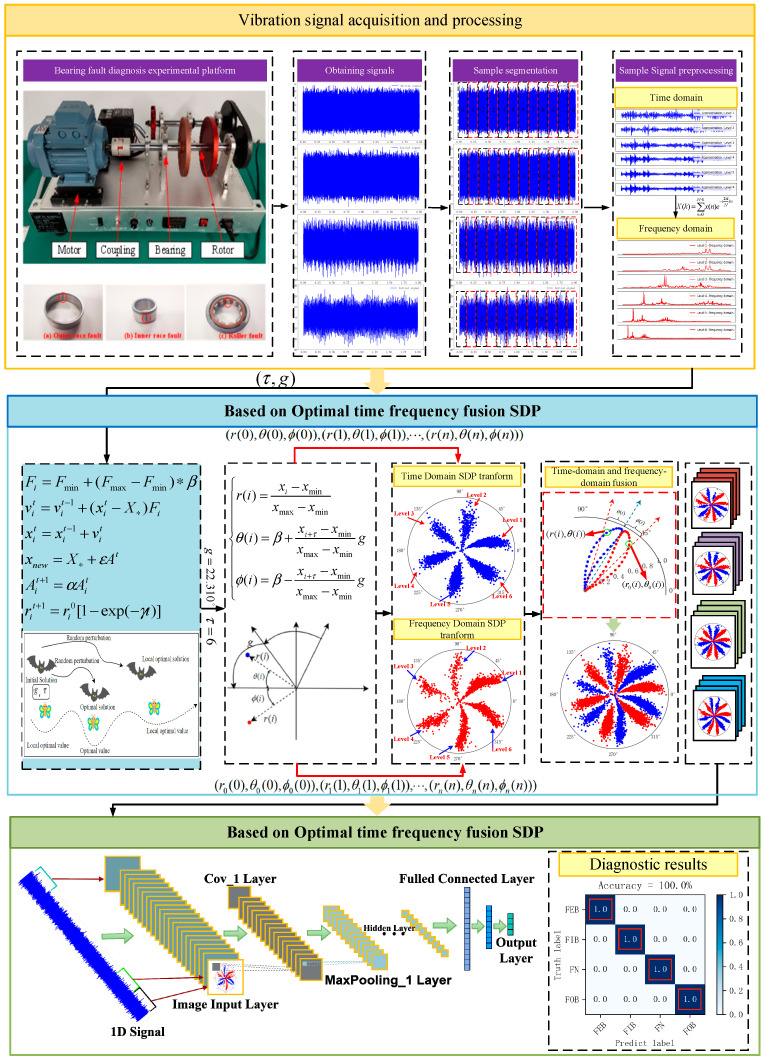
Proposed method framework.

This paper proposes using a 1D to 2D optimal time frequency fusion SDP method to enhance bearing fault signal features. This method resolves difficulties in parameter selection, the lack of multi-scale analysis for complex signals, high computational complexity, and achieves enhanced bearing fault features. Figure 7 illustrates the overall architecture based on optimal time frequency fusion SDP bearing fault feature enhancement and diagnosis method. The specific steps are as follows:

- Vibration signal of normal and fault bearing states are acquired using acceleration sensors.
- The vibration signal is high-pass filtered, and segmented into samples.
- The wavelet basis function ‘db1’ is used to decompose these samples six times. The wavelet decomposition high frequency part captures the signal’s fast changes and features, which will be used as the signal input for the optimal time frequency fusion SDP.
- Adaptively obtain the time-delay parameter and expansion factor of the optimal time frequency fusion SDP image by bat algorithm.
- The optimal time frequency fusion SDP method is used to transform the sample signal into a 2D image.
- Divide the 2D images into a training dataset and a testing dataset. Construct a DCNN model and input the training dataset into the model for training.
- Establish the diagnostic model and input the testing dataset for testing, then, after the training and the testing, obtain the diagnostic results.

## 4. Experimental Verification

### 4.1. Case 1: Dataset I

**Figure 8:**
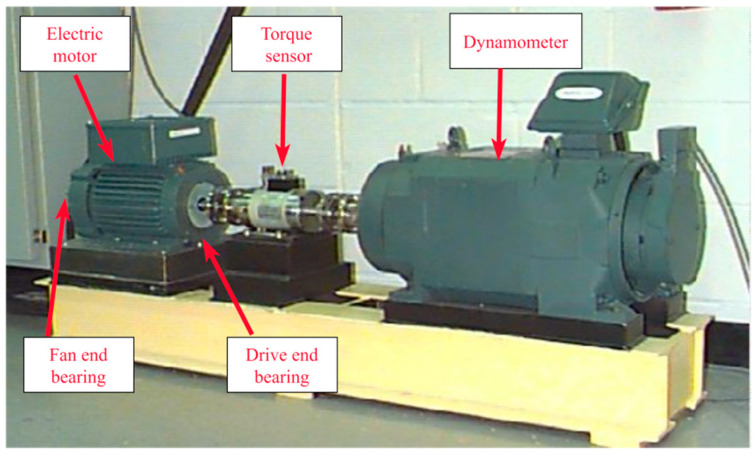
Experimental equipment.

The Case Western Reserve University (CWRU) bearing fault dataset is a publicly available dataset widely used in bearing fault diagnosis. It covers vibration data for bearings in various fault states and under different operating conditions. Figure 8 illustrates the experimental setup used for data collection, which includes a 2 HP motor, torque sensor, dynamometer, control electronics, and bearings [28]. The vibration signals were collected using a 2 Hp motor as the drive system, with bearings mounted both away from and close to the motor drive. Sensors were placed at the drive and fan ends, with a sampling frequency of 12 kHz or 48 kHz. The dataset includes vibration data from the drive end, fan end, and pedestal. The experiments were conducted under various engine load conditions: 0 HP, 1 HP, 2 HP, and 3 HP, corresponding to speeds of 1797 rpm, 1772 rpm, 1750 rpm, and 1730 rpm, respectively. The bearing faults resulting from EDM forming included outer race, roller, and inner race faults, each state having three different fault diameters (0.007 inches, 0.014 inches, and 0.021 inches).

#### 4.1.1. Signal Description and Processing

**Figure 9:**
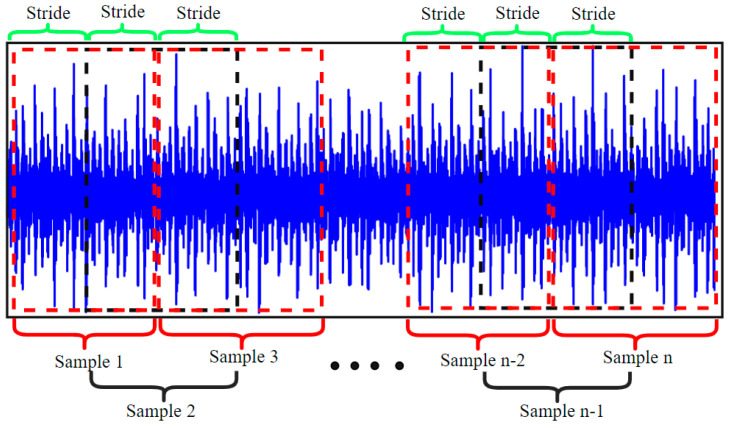
Signal overlap sampling.

This paper analyses vibration signals based on 10 types of fault state for rolling bearings collected under working conditions involving a motor load of 0 and a speed of 1797 rpm, as illustrated in Table 1. The dataset comprises 1 normal state and 9 fault states, representing 3 distinct faults: inner race fault, roller fault, and outer race fault, with each state having 3 fault diameters (0.007 inches, 0.014 inches, and 0.021 inches). The signal overlap sampling method for a bearing’s different state data is used in this paper to overcome the insufficient data volume problem, as illustrated in schematic Figure 9. Overlap sampling is first performed with a sample length of 8192 data points, resulting in a total sample length of 100 samples. Similarly, 100 samples are acquired for each bearing state, with the dataset illustrated in Table 1.

#### 4.1.2. Optimal Time Frequency Fusion SDP Parameter Selection Method

**Figure 10:**
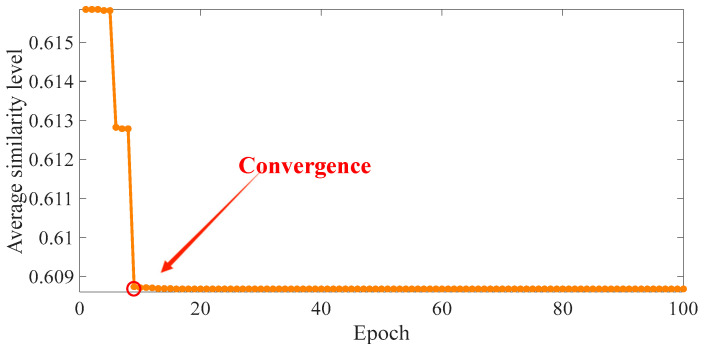
Change in average similarity.

The optimal τ and g values are sought by the bat algorithm. The parameters of the bat algorithm are set as follows: *g* ranges from $\left[20^{{}^{\circ}}\;to\;60^{{}^{\circ}}\right]$, τ ranges from [1 to 10], the number of bats in the bat population is 10, the number of iterations is 100, and the maximum and minimum values of the frequency are set to 2 and 0, respectively. The loudness is 0.5, the pulse rate is 0.5, and the fitness function is referred to in Formulas (6)–(9). Then, 100 iterations are performed. The final outputs are *g =* $30.165^{{}^{\circ}}$ and τ = 9, and the average similarity of comparison between images is 0.608. The fitness function is illustrated in Figure 10.

#### 4.1.3. Optimal Time Frequency Fusion SDP Feature Extraction Method

**Figure 11:**
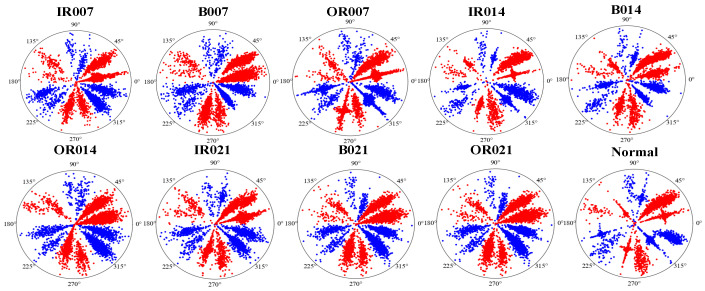
Optimal time frequency fusion SDP transformation results based on dataset I.

The vibration signal of bearings in different states was transformed into 2D images using the optimal time frequency fusion SDP method, which combines selected expansion factors and time-delay parameters. Figure 11 illustrates the differences between the optimal time frequency fusion SDP images of bearings in different states. These differences are mainly expressed in the saturation level of the petals and the sparseness of the points on the petals. By transforming optimal time frequency fusion SDP images, the image features of bearings in different states can be visually represented, enhancing the accuracy of fault diagnosis.

#### 4.1.4. Diagnosis Results

**Figure 12:**
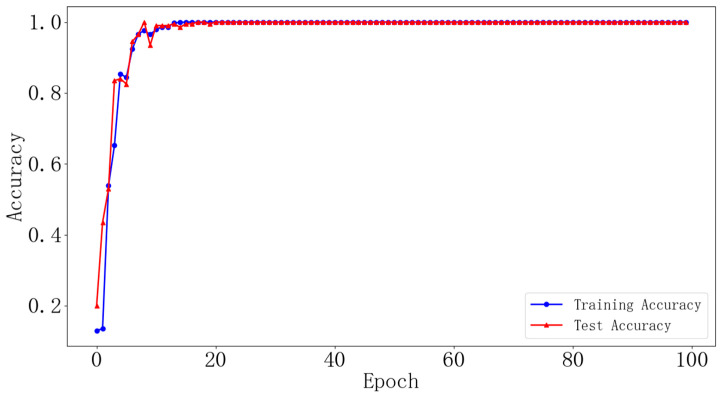
Changes in network training accuracy (dataset I).

**Figure 13:**
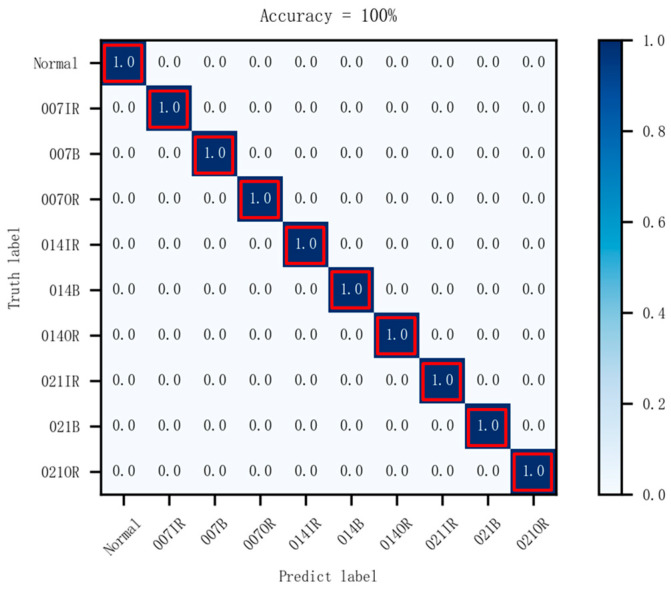
Confusion matrix (dataset I).

The vibration signals from bearings in different states were transformed using optimal time frequency fusion SDP. The training dataset and testing dataset were distributed in the ratio 4:1, that is, 80 samples for each bearing state for the training dataset and 20 samples for the testing dataset. The DCNN model was used to classify the bearing state based on the provided dataset. The model learning rate is set to 0.001 and the iteration is 100. Figure 12 illustrates the change in training accuracy between the training dataset and testing dataset during the experiment. Furthermore, the testing results are illustrated in Figure 13.

Figure 12 illustrates the accuracy change in the training datasets and testing datasets after 100 iterations. Figure 12 illustrates that, during the training process, there are large fluctuations in the accuracy of the training dataset and testing dataset at the initial stage. After approximately the 18th iteration, these fluctuations gradually converge, and the accuracy of the training dataset and testing dataset stabilizes at 99% and eventually reaches 100%. The results demonstrate the strong convergence ability of the proposed method in this paper. In addition, Figure 13 presents the prediction results of the model for ten different states of the bearings. It can be seen that the accuracy of the bearing fault diagnosis model in ten different states is 100%. The results demonstrate the effectiveness of the proposed method in this paper for bearing fault diagnosis.

### 4.2. Case 2: Dataset II

#### 4.2.1. Signal Description and Processing

**Figure 14:**
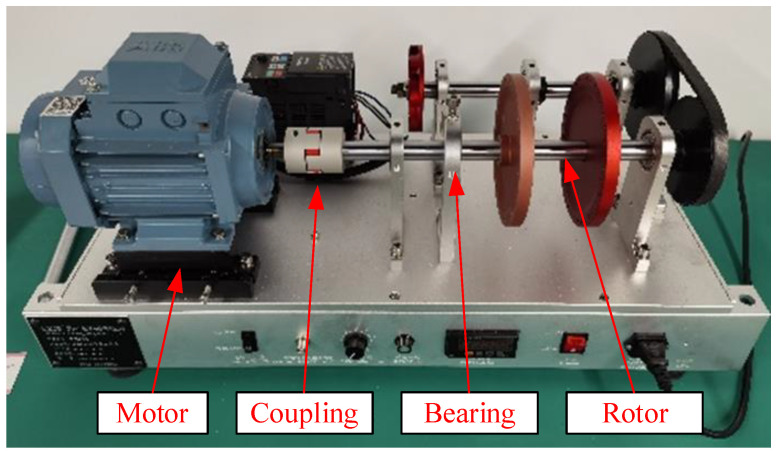
Experimental device.

**Figure 15:**
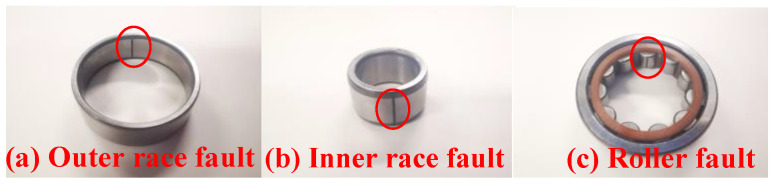
Types of bearing faults: (**a**) outer race fault, (**b**) inner race fault, and (**c**) roller fault.

The bearing vibration signals from the laboratory experimental device were acquired by an acceleration sensor with a sampling frequency of 100 kHz, while the experimental speed was 1500 rpm. The experimental device consists of a motor, a connection device, a bearing, and a rotor. Its structure is schematically illustrated in Figure 14. To simulate different bearing fault states, three types of bearings with different faults are used: a bearing with an outer race fault, a bearing with an inner race fault, and a bearing with a roller fault. The schematic diagrams of various bearing fault states are depicted in Figure 15.

The experimentally acquired data of different bearing states were processed as follows: firstly, the data from different states of the bearing were sampled. Sampling occurred every 16,384 data points, resulting in 100 samples. Then, these samples were subjected to signal pre-processing. The dataset description is presented in Table 2.

#### 4.2.2. Optimal Time Frequency Fusion SDP Parameter Selection and Feature Extraction Method

**Figure 16:**
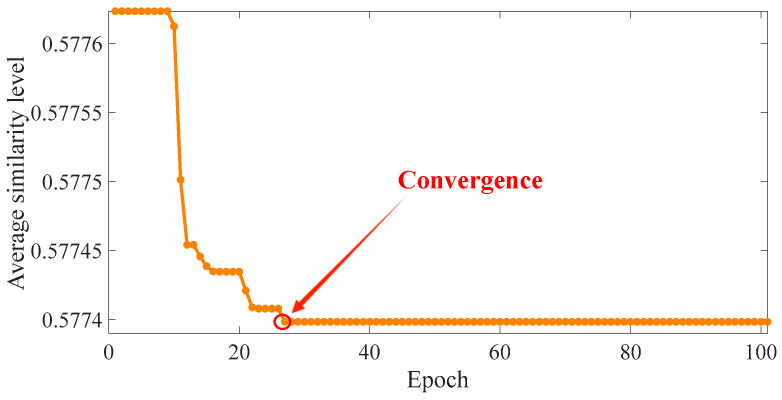
Value of fitness function.

The optimal τ and *g* values are obtained by the bat algorithm. The parameters of the bat algorithm are set as follows: *g* ranges from $\left[20^{{}^{\circ}}\;to\;60^{{}^{\circ}}\right]$, τ ranges from [1 to 10], the number of bats in the bat population is 10, the number of iterations is 100, the maximum and minimum values of the frequency are set to 2 and 0, respectively, the loudness is 0.5, the pulse rate is 0.5, and the fitness function is referred to in Formulas (6)–(9). Then, 100 iterations are performed. The final outputs are *g =* $22.310^{{}^{\circ}}$ and τ = 6, and the average similarity of comparison between images is 0.577. The fitness function is illustrated in Figure 16.

**Figure 17:**
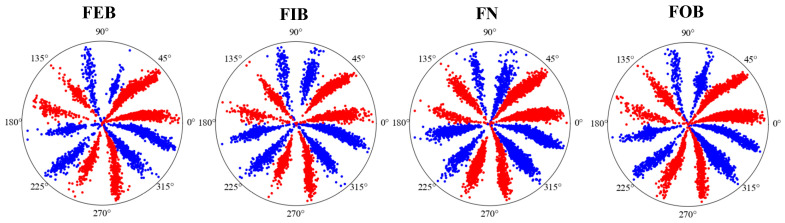
Optimal time frequency fusion SDP transformation results based on dataset II.

Figure 17 gives the optimal time frequency fusion SDP method images of four different states of the bearing when *g* = $22.310^{{}^{\circ}}\;and\;\tau$ = 6. As can be seen from Figure 17, the optimal time frequency fusion SDP images of the different bearing states illustrates obvious differences, mainly in the saturation degree of the petals and the sparseness of the points on the petals. Therefore, by drawing optimal time frequency fusion SDP images, the signal features of the bearings in different states can be intuitively expressed, so as to achieve accurate fault diagnosis.

#### 4.2.3. Diagnosis Results

**Figure 18:**
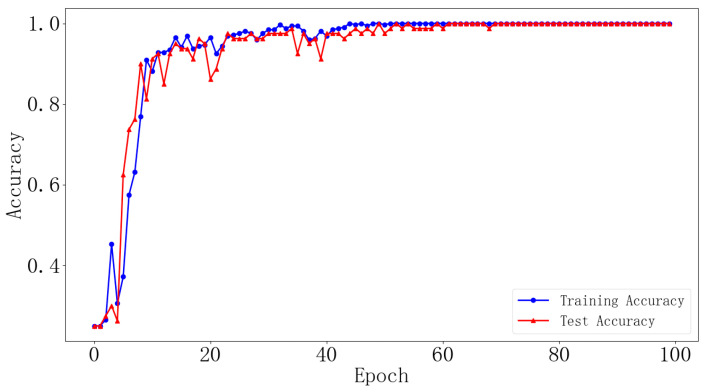
Changes in network training accuracy (dataset II).

**Figure 19:**
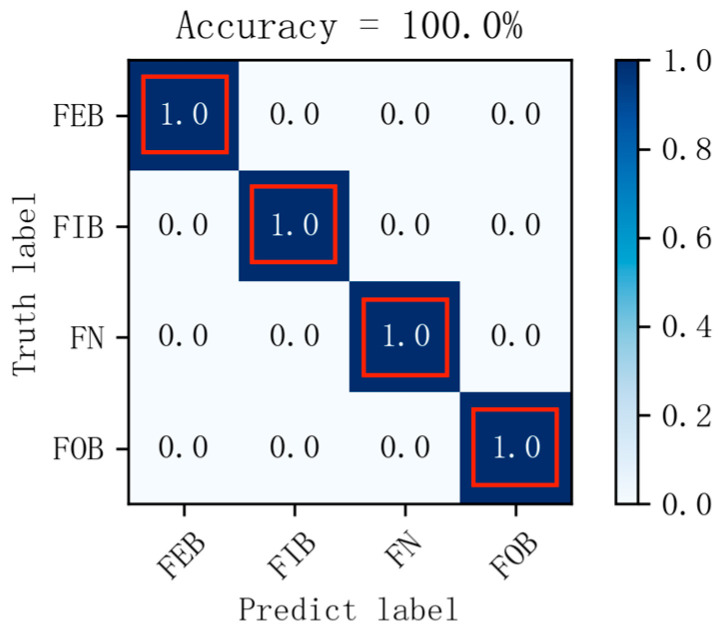
Confusion matrix (dataset II).

The experimentally acquired signals of the different bearing states are transformed using the optimal time frequency fusion method for 400 samples. Each bearing state divides the dataset into a training dataset and a testing dataset in the ratio 4:1. The model learning rate is set to 0.001 and the iteration is 100. The dataset is used as an input of the DCNN for fault classification. The variation in training accuracy obtained from the experiment is illustrated in Figure 18, and the diagnostic results of the testing dataset can be illustrated in Figure 19.

Figure 18 illustrates the change in accuracy of the training dataset and testing dataset after 100 iterations. From Figure 18, during the training process, there are significant fluctuations in the accuracy of the training dataset and testing dataset in the early stages. After approximately the 60th iteration, the fluctuation gradually decreases, and the accuracies of the training dataset and testing dataset stabilize. In addition, Figure 19 illustrates the prediction results of the model for four different states of the bearing. Figure 19 illustrates that the model’s prediction accuracy for four different states of the bearing reaches 100%.

## 5. Comparison Experiment

### 5.1. Comparison of 1D Signal to 2D Image Transformation Methods

**Figure 20:**
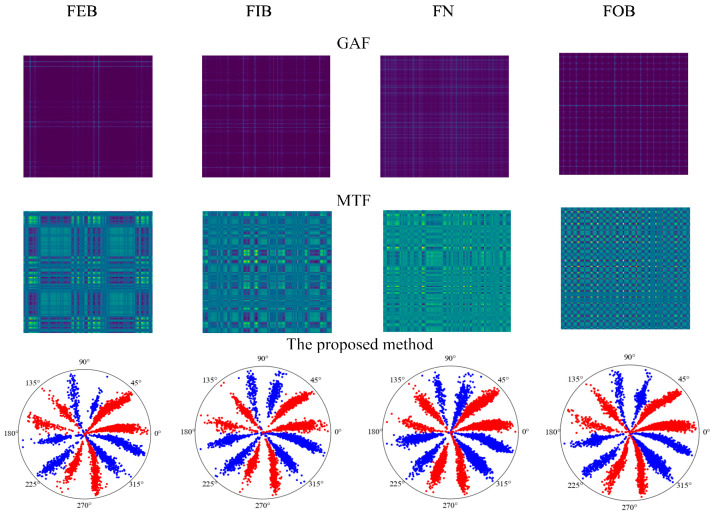
Transformation results of different methods based on dataset II.

**Figure 21:**
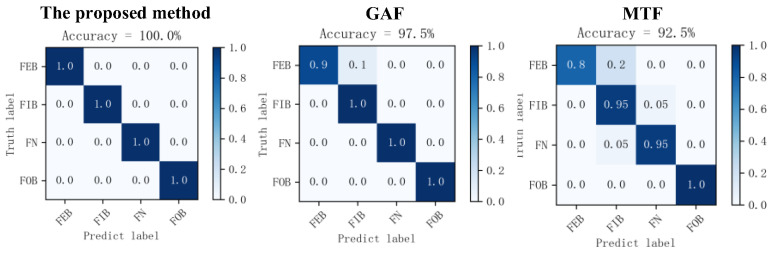
Confusion matrix of different methods’ diagnostic results.

To verify the effectiveness of the method proposed in this paper more comprehensively, the optimal time frequency fusion SDP transformation method is compared with the Markov transition field (MTF) and Gramian angular field (GAF) methods in this section. The specific steps are as follows:

- The samples acquired from various bearing states were transformed into 2D images using the optimal time frequency fusion SDP method transformation technique, including the Markov transition field (MTF) and Gramian angular field (GAF) methods. Subsequently, they were split into a training dataset and testing dataset in the ratio 4:1. Figure 20 displays the 2D images obtained using various methods, while Table 3 illustrates the dataset operation time of these methods.

- The 2D image obtained from the transformation of MTF, GAF, and optimal time frequency fusion SDP methods is input into the DCNN. The network performs adaptive learning of image features and classification, with the learning rate set to 0.001 and the number of iterations to 100.
- The training time of the dataset, training accuracy, etc., for the various image transformation methods are presented in Table 4. The diagnostic accuracy of the different methods is illustrated through a confusion matrix, as illustrated in Figure 21.

As seen in Table 3 and Table 4, the method proposed in this paper outperforms the other two methods in terms of transformation time, accuracy, and epoch of reach of the convergence. Regarding image transformation, the optimal time frequency fusion SDP transformation method produces an image in 0.111 s, which is superior in terms of real-time performance and computational efficiency compared to the other two methods. Training accuracy is improved by 7.5% compared to the MTF method and by 2.5% compared to GAF. In addition, the method described in this paper is significantly superior to the other two methods regarding the epoch number of reach of the convergence. Therefore, under the same conditions, the method proposed in this paper has a shorter training time than GAF and MTF. It enables faster model training and quicker fault diagnosis.

Therefore, the method proposed in this paper is more computationally efficient and better in real-time than the other two methods, further validating its feasibility and superiority.

### 5.2. Comparison of the Proposed Method with Traditional SDP Methods

**Figure 22:**
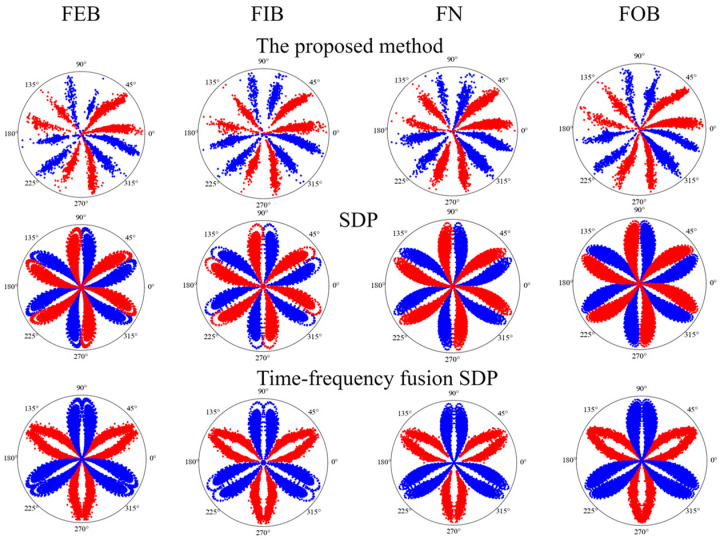
Transformation results of different SDP methods based on dataset II.

**Figure 23:**
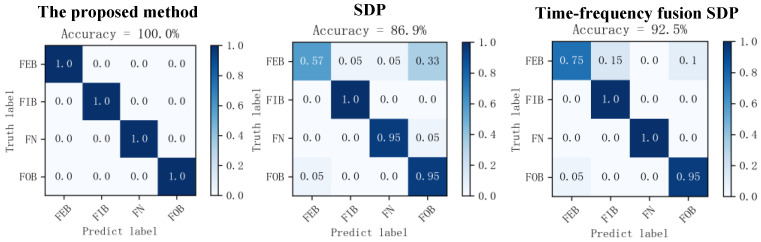
Confusion matrix of different SDP methods’ diagnostic results.

To further validate the excellent performance of the proposed method in this paper in terms of signal feature enhancement, the process is compared with the traditional SDP image transformation method and the time frequency fusion SDP method. The specific steps are as follows:

- Using the methods proposed in this paper, the traditional SDP image transformation method and the time frequency fusion SDP method, the acquired signal of four different states of the bearing are transformed into 2D images, respectively. Then, they are divided into a training dataset and a testing dataset according to the ratio 4:1. Figure 22 illustrates the 2D images obtained using the different methods.
- The SDP image dataset, the time frequency fusion SDP image dataset, and the optimal time frequency fusion SDP image dataset are input into the DCNN. The network performs adaptive learning of the image features and classification. The network learning rate is set to 0.001, and the number of iterations is set to 100.
- The diagnostic results of the different SDP methods are presented in Table 5 and the training effect is visualized as a confusion matrix, as illustrated in Figure 23.

As illustrated in Table 5, the method proposed in this paper outperforms the other two methods in terms of accuracy and the number of training rounds. Regarding training accuracy, the accuracy is enhanced by 13.1% compared to the traditional SDP method and 7.5% compared to the time frequency fusion SDP method. In addition, the method described in this paper is significantly superior to the other two methods regarding the number of training epochs needed to achieve the highest accuracy. Therefore, under the same conditions, this paper’s method has a shorter training time than the traditional SDP and the time frequency fusion SDP methods. It enables faster model training and fault diagnosis.

## 6. Conclusions

Aiming to address the challenges of strong background noise submerging bearing fault features, and the fact of there being limited information in 1D signals from bearings, this paper introduces an optimal time frequency fusion SDP method. This method combines a signal’s time and frequency domains with varying scales on the SDP image to enhance the signal distinctions between different bearing states. This approach enables the precise identification of bearing faults. The experiments were validated using a public dataset and laboratory data. The results illustrate that the fault diagnosis accuracy reached 100%. The validity of the proposed method is also verified through comparative analysis experiments. The method proposed in this paper provides an innovative two-stage model for bearing fault diagnosis that combines offline training and online testing, which is of significant engineering research value.

In addition, the approach investigated in this paper is applicable to six-segment time-domain and six-segment frequency-domain signal inputs. Future research will explore the applicability of this approach to other decomposition algorithms, as well as its effectiveness in rotating machinery fault diagnosis. These aspects will be addressed in subsequent research.

## Figures and Tables

**Table 1:** Description of dataset I.

Bearing State	Fault Diameter (Inches)	Number of Samples	Label
Normal	-	100	Normal
Inner Race fault	0.007	100	IR007
Inner Race fault	0.014	100	IR014
Inner Race fault	0.021	100	IR021
Roller fault	0.007	100	B007
Roller fault	0.014	100	B014
Roller fault	0.021	100	B021
Outer Race fault	0.007	100	OR007
Outer Race fault	0.014	100	OR014
Outer Race fault	0.021	100	OR021

**Table 2:** Description of dataset II.

Bearing State	Number of Samples	Label
Normal	100	FN
Outer Race fault	100	FOB
Inner Race fault	100	FIN
Roller fault	100	FEB

**Table 3:** Dataset production time for different methods.

Methods	Average Image Transformation Time (s)	Dataset Operation Time (s)
GAF	0.692	277.070
MTF	0.381	152.519
The proposed method	0.111	44.605

**Table 4:** Diagnostic results of different methods.

Methods	Classifier	Accuracy (%)	Train Time (s)	Epoch of Reach the Convergence
GAF	DCNN	97.5	1326	34
MTF	DCNN	93.8	1329	36
The proposed method	DCNN	100	265	23

**Table 5:** Diagnostic results of the different SDP methods.

Methods	Classifier	Accuracy (%)	Epoch of Reach the Convergence
Time frequency fusion SDP	DCNN	92.5	76
SDP	DCNN	86.9	84
The proposed method	DCNN	100	23

## Data Availability

The CWRU data can be found at https://engineering.case.edu/bearingdatacenter/download-data-file (accessed on 21 April 2024).
